# Supplementary material for: The incidence, characteristics and outcomes of pregnant women hospitalized with symptomatic and asymptomatic SARS-CoV-2 infection in the UK from March to September 2020: A national cohort study using the UK Obstetric Surveillance System (UKOSS)
Source: PLoS One. 2021 May 5;16(5):e0251123. doi: 10.1371/journal.pone.0251123 (PMC8099130; doi:10.1371/journal.pone.0251123)
Supplement: S1 Table — (DOCX) [file pone.0251123.s001.docx]

**S1 Table. Relevant comorbidities**

| **Any Relevant Comorbidity** |
| --- |
| **Conditions on current NHS shielding patient list**   - Solid organ transplant recipient on long term immune suppression treatment - Cancers with active chemotherapy or immunosuppressive treatments /Blood/bone marrow cancer at any treatment stage - Immunosuppression sufficiently increasing infection risk - Severe respiratory disease e.g., Severe asthma (≥3 prescribed courses of steroids in preceding 12 months) - Rare diseases or inborn errors of metabolism e.g., Homozygous sickle cell disease - Significant heart disease   **Conditions moderately associated with increased risk of complications as per current NHS guidance**   - Chronic, non-severe respiratory disease e.g., Asthma - Chronic kidney disease (CKD) - Chronic cardiac disease - Chronic liver disease e.g., Chronic infective hepatitis - Chronic neurological conditions: Epilepsy - Diabetes mellitus: Type1 or Type2 - Conditions or treatments that predispose to infection (e.g., steroid treatment): e.g., Systemic lupus erythematosus, Inflammatory bowel disease   **Other medical conditions that investigators hypothesized could elevate risk**   - Osteoporosis - Cardiac disease e.g., Arrythmias - Treated hypertension - Hyperthyroidism - Cirrhosis (if not above, e.g., non-alcoholic fatty liver disease) - Malabsorption e.g., Coeliac disease or Peptic ulcer (gastric or duodenal) - Severe mental illness: e.g., Bipolar affective disorder, Psychosis, Schizophrenia or schizoaffective disorder - HIV infection - History of venous thromboembolism - Tuberculosis |
