# Supplementary material for: The incidence, characteristics and outcomes of pregnant women hospitalized with symptomatic and asymptomatic SARS-CoV-2 infection in the UK from March to September 2020: A national cohort study using the UK Obstetric Surveillance System (UKOSS)
Source: PLoS One. 2021 May 5;16(5):e0251123. doi: 10.1371/journal.pone.0251123 (PMC8099130; doi:10.1371/journal.pone.0251123)
Supplement: S2 Table — (DOCX) [file pone.0251123.s002.docx]

**S2 Table. Ethnic group based on UK Census Coding**

| Ethnic Group |  |
| --- | --- |
| White | British |
|  | Irish |
|  | Any other white background |
| Mixed | White and black Caribbean |
|  | White and black African |
|  | White and Asian |
|  | Any other mixed background |
| Asian or Asian British | Indian |
|  | Pakistani |
|  | Bangladeshi |
|  | Any other Asian background |
| Black or Black British | Caribbean |
|  | African |
|  | Any other black background |
| Chinese |  |
| Any other ethnic group |  |
