# Supplementary material for: The incidence, characteristics and outcomes of pregnant women hospitalized with symptomatic and asymptomatic SARS-CoV-2 infection in the UK from March to September 2020: A national cohort study using the UK Obstetric Surveillance System (UKOSS)
Source: PLoS One. 2021 May 5;16(5):e0251123. doi: 10.1371/journal.pone.0251123 (PMC8099130; doi:10.1371/journal.pone.0251123)
Supplement: S3 Table — (DOCX) [file pone.0251123.s003.docx]

**S3 Table. Sensitivity analysis for pre-existing medical comorbidities in women with symptomatic SARS-CoV-2 infection**

|  | Women with symptomatic SARS-CoV-2 (N=1148) | Historical comparison cohort (N=694) | OR (95% CI) | aOR* |
| --- | --- | --- | --- | --- |
| Any relevant pre-existing medical problems | 156 (22%) | 90 (13%) | 1.85 (1.39-2.46)  p<0.001 | 1.83 (1.32-2.54)  p<0.001 |
| Asthma | 49 (7%) | 31 (4%) | 1.56 (0.98-2.47)  p=0.060 | 2.12 (1.25-3.58)  p=0.005 |
| Hypertension | 24 (3%) | 3 (<1%) | 7.92 (2.37-26.42)  p=0.001 | 3.63 (0.99-13.30)  p=0.051 |
| Cardiac disease | 13 (2%) | 10 (1%) | 1.25 (0.54-2.88)  p=0.593 | 1.47 (0.59-3.63)  p=0.403 |
| Diabetes | 22 (3%) | 7 (1%) | 3.08 (1.31-7.27)  p=0.010 | 1.34 (0.48-3.68)  p=0.570 |
| Liver Conditions | 12 (1%) | 5 (1%) | 1.46 (0.51-4.15)  p=0.482 | 1.29 (0.35-4.72)  p=0.692 |
| Renal Conditions | 9 (1%) | 5 (1%) | 1.08 (0.36-3.26)  p=0.879 | 1.16 (0.28-4.80)  p=0.828 |

***Adjusted for ethnicity, BMI, smoking, woman’s age**
