# Supplementary material for: The incidence, characteristics and outcomes of pregnant women hospitalized with symptomatic and asymptomatic SARS-CoV-2 infection in the UK from March to September 2020: A national cohort study using the UK Obstetric Surveillance System (UKOSS)
Source: PLoS One. 2021 May 5;16(5):e0251123. doi: 10.1371/journal.pone.0251123 (PMC8099130; doi:10.1371/journal.pone.0251123)
Supplement: S4 Table — (DOCX) [file pone.0251123.s004.docx]

**S4 Table. Characteristics of pregnant women with confirmed SARS-CoV-2 infection admitted to hospital in the UK compared to a historical cohort without SARS-CoV-2**

| Characteristic | Women with SARS-CoV-2 (N=1148) | Historical comparison cohort (N=694) | OR (95% CI) | aOR** |
| --- | --- | --- | --- | --- |
|  | **Number (%) *** | **Number (%) *** |  |  |
| Age (years): |  |  |  |  |
| <20 | 23 (2%) | 18 (3%) | 0.80 (0.43 – 1.49)  p=0.480 | 1.37 (0.67-2.78)  p=0.384 |
| 20-34 | 764(67%) | 477(69%) | 1 (base) | 1 |
| ≥35 | 360 (31%) | 199 (29%) | 1.13 (0.92-1.39)  p=0.250 | 0.94 (0.74-1.19)  p=0.605 |
| Missing | 1 | 0 | - | - |
| Body Mass index (BMI): |  |  |  |  |
| Normal | 409 (37%) | 337 (50%) | 1 | 1 |
| Overweight | 348 (31%) | 181 (27%) | 1.58 (1.26-1.99)  p<0.001 | 1.52 (1.18-1.95)  p=0.001 |
| Obese | 345 (31%) | 155 (23%) | 1.83 (1.45 - 2.33)  p<0.001 | 1.75 (1.33-2.27)  p<0.001 |
| Missing | 43 | 18 | - | - |
| Either woman or partner in paid work | 897 (78%) | 537 (77%) | 1.04 (0.84 – 1.31)  p=0.704 | Omitted |
| Ethnic Group |  |  |  |  |
| White | 594 (53%) | 558 (81%) | 1 | 1 |
| Asian | 294 (26%) | 79 (11%) | 3.50 (2.66-4.60)  p<0.001 | 3.38 (2.53-4.52)  p<0.001 |
| Black | 155 (14%) | 26 (4%) | 5.60 (3.64 – 8.62)  p<0.001 | 4.81 (3.09-7.49)  p<0.001 |
| Chinese | 12 (1%) | 7 (1%) | 1.61 (0.63– 4.11)  p=0.320 | 1.59 (0.60-4.18)  p=0.350 |
| Other | 52 (5%) | 5 (1%) | 9.77 (3.87-24.64)  p<0.001 | 9.85 (3.85-25.15)  p<0.001 |
| Mixed | 20 (2%) | 14 (2%) | 1.34 (0.67-2.68)  p=0.405 | 1.36 (0.66-2.81)  p=0.402 |
| Missing | 21 | 5 |  |  |
| Current smoking | 99 (10%) | 135 (20%) | 0.43 (0.32-0.57)  p<0.001 | 0.58 (0.43-0.79)  p<0.001 |
| Missing | 109 | 10 |  |  |
| Any relevant pre-existing medical problems | 220 (19%) | 90 (13%) | 1.59 (1.22-2.08)  p<0.001 | 1.64 (1.22-2.20)  p=0.001 |
| Asthma | 77 (7%) | 31 (4%) | 1.54 (1.00-2.36)  p=0.049 | - |
| Hypertension | 26 (2%) | 3 (<1%) | 5.33 (1.61 – 17.70)  p=0.006 | - |
| Cardiac disease | 21 (2%) | 10 (1%) | 1.27 (0.60-2.72)  p=0.531 | - |
| Diabetes | 28 (2%) | 7 (1%) | 2.45 (1.07-5.65)  p=0.035 | - |
| Multiparous | 675 (59%) | 420 (61%) | 0.95 (0.78-1.15)  p=0.595 | Omitted |
| Missing | 9 | 0 |  | - |
| Multiple pregnancy | 16 (1%) | 13 (2%) | 0.74 (0.35-1.55)  p=0.425 | Omitted |
| Gestational diabetes | 116 (10%) | 37 (5%) | 2.00 (1.36-2.93)  p<0.001 | Omitted |
| Gestation at diagnosis (weeks) |  |  |  |  |
| <22 | 60 (5%) |  |  |  |
| 22-27 | 74 (6%) |  |  |  |
| 28-31 | 106 (9%) |  |  |  |
| 32-36 | 166(15%) |  |  |  |
| 37 or more | 295 (26%) |  |  |  |
| Peripartum | 438 (38%) |  |  |  |
| Missing | 6 |  |  |  |

* Percentages of those with complete data

** adjusted for ethnicity, BMI, Any previous medical problem, Smoking
