# Supplementary material for: The incidence, characteristics and outcomes of pregnant women hospitalized with symptomatic and asymptomatic SARS-CoV-2 infection in the UK from March to September 2020: A national cohort study using the UK Obstetric Surveillance System (UKOSS)
Source: PLoS One. 2021 May 5;16(5):e0251123. doi: 10.1371/journal.pone.0251123 (PMC8099130; doi:10.1371/journal.pone.0251123)
Supplement: S5 Table — (DOCX) [file pone.0251123.s005.docx]

**S5 Table. Characteristics of pregnant women with symptomatic versus asymptomatic SARS-CoV-2 hospitalized in the UK**

| Characteristic | Women with symptomatic SARS-CoV-2 (N=722) | Women with asymptomatic SARS-CoV-2 (N=426) | OR (95% CI) | aOR** |
| --- | --- | --- | --- | --- |
|  | Number (%) * | Number (%) * |  |  |
| Age (years): |  |  |  |  |
| <20 | 12 (2%) | 11 (3%) | 0.76 (0.33-1.74)  p=0.512 | **1.02 (0.40-2.65)**  **p=0.395** |
| 20-34 | 451 (62%) | 313 (74%) | 1 | **1** |
| ≥35 | 258 (36%) | 102 (24%) | 1.75 (1.34-2.30)  p<0.001 | **1.62 (1.18-2.22)**  **p=0.003** |
| Missing | 1 | 0 | - | **-** |
| Body Mass index (BMI): |  |  | 1.05 (1.03-1.07) |  |
| Normal | 221 (32%) | 188 (46%) | 1 | **1** |
| Overweight | 237 (34%) | 111 (27%) | 1.82 (1.35-2.45)  p<0.001 | **1.66 (1.19-2.31)**  **p<0.001** |
| Obese | 235 (34%) | 110 (27%) | 1.82 (1.35-2.45)  p<0.001 | **1.72 (1.22-2.41)**  **p<0.001** |
| Missing | 27 | 16 | - | **-** |
| Either woman or partner in paid work | 574 (80%) | 323 (76%) | 1.24 (0.93-1.65)  p=0.145 | **Omitted** |
| Ethnic Group |  |  |  |  |
| White | 318 (44%) | 276 (65%) | 1 | **1** |
| Asian | 208 (30%) | 84 (20%) | 2.17 (1.61-2.93)  p<0.001 | **2.22 (1.58-3.12)**  **p<0.001** |
| Black | 122 (17%) | 33 (8%) | 3.20 (2.11-4.87)  p<0.001 | **2.45 (1.55-3.87)**  **p<0.001** |
| Chinese | 8 (1%) | 4 (1%) | 1.74 (0.52-5.83)  p=0.372 | **1.80 (0.46-7.02)**  **p=0.399** |
| Other | 36 (5%) | 16 (4%) | 1.95 (1.06-3.60)  p=0.032 | **2.19 (1.08-4.41)**  **p=0.029** |
| Mixed | 15 (2%) | 5 (1%) | 2.60 (0.93-7.26)  p=0.067 | **1.72 (0.58-5.02)**  **p=0.321** |
| Missing | 13 | 8 | - | **-** |
| Current smoking | 42 (6%) | 57 (16%) | 0.34 (0.22-0.51)  p<0.001 | **0.46 (0.29-0.72)**  **p=0.001** |
| Missing | 35 | 74 | - | **-** |
| Any Relevant Pre-existing medical problems | 156 (22%) | 64 (15%) | 1.56 (1.13-2.15)  p=0.006 | **1.38 (0.96-1.99)**  **p=0.079** |
| Multiparous | 436 (61%) | 239 (57%) | 1.18 (0.92-1.50)  p=0.190 | **Omitted** |
| Missing | 4 | 5 | - | **-** |
| Multiple pregnancy | 12 (2%) | 4 (1%) | 1.78 (0.57-5.56)  p=0.319 | **Omitted** |
| Gestational diabetes | 76 (11%) | 40 (9%) | 1.13 (0.76-1.70)  p=0.537 | **Omitted** |
| Gestation at diagnosis (weeks) |  |  |  |  |
| <22 | 43 (6%) | 17 (4%) | - | **-** |
| 22-27 | 67 (9%) | 10 (2%) | - | **-** |
| 28-31 | 98 (14%) | 8 (2%) | - | **-** |
| 32-36 | 127 (18%) | 39 (9%) | - | **-** |
| 37 or more | 131 (18%) | 164 (39%) | - | **-** |
| Peripartum | 251 (35%) | 187 (44%) | - | **-** |
| Missing | 5 | 1 | - | **-** |

* Percentages of those with complete data

** adjusted for ethnicity, BMI, Any previous medical problem, Smoking
