# Supplementary material for: The incidence, characteristics and outcomes of pregnant women hospitalized with symptomatic and asymptomatic SARS-CoV-2 infection in the UK from March to September 2020: A national cohort study using the UK Obstetric Surveillance System (UKOSS)
Source: PLoS One. 2021 May 5;16(5):e0251123. doi: 10.1371/journal.pone.0251123 (PMC8099130; doi:10.1371/journal.pone.0251123)
Supplement: S6 Table — (DOCX) [file pone.0251123.s006.docx]

**S6 Table. Maternal and perinatal outcomes and diagnoses amongst women with confirmed SARS-CoV-2 infection in pregnancy admitted to intensive care**

| Maternal outcomes | Women with SARS-CoV-2 admitted to intensive care (N=63) |
| --- | --- |
|  | **Number (%)** |
| Required ECMO | 4 (6%) |
| SARS-CoV-2 pneumonia | 47 (75%) |
| Died | 5 (8%) |
| Discharged well | 56 (92%) |
| Missing outcome information | 2 |
| Multiple pregnancy | 2 (3%) |
| Ongoing pregnancy | 3 (5%) |
| Pregnancy completed | 60 (95%) |
| Pregnancy loss | 1 (2%) |
|  | **Infant outcomes (N=61)** |
| Stillbirth | 1 (2%) |
| Live birth | 60 (98%) |
| Neonatal death | 1 (2%) |
| Gestation at end of pregnancy (weeks) |  |
| 22-27 | 5 (8%) |
| 28-31 | 12 (20%) |
| 32-36 | 21 (36%) |
| 37 or more | 21 (36%) |
| Missing | 1 |
