# Supplementary material for: The incidence, characteristics and outcomes of pregnant women hospitalized with symptomatic and asymptomatic SARS-CoV-2 infection in the UK from March to September 2020: A national cohort study using the UK Obstetric Surveillance System (UKOSS)
Source: PLoS One. 2021 May 5;16(5):e0251123. doi: 10.1371/journal.pone.0251123 (PMC8099130; doi:10.1371/journal.pone.0251123)
Supplement: S7 Table — (DOCX) [file pone.0251123.s007.docx]

**S7 Table. Indication for cesarean in women with confirmed symptomatic, asymptomatic SARS-CoV-2 and a historical comparison cohort**

| Indication for cesarean | Women with symptomatic SARS-CoV-2 | Women with asymptomatic SARS-CoV-2 | Historical comparison cohort |
| --- | --- | --- | --- |
| Maternal compromise due to SARS-CoV-2 | 64 (20) |  | - |
| Failure to progress | 67 (21) | 34 (22) | 39 (19) |
| Fetal Indication | 71 (23) | 35 (23) | 51 (25) |
| Maternal Request | 13 (4) | 6 (4) | 12 (6) |
| Previous Cesarean | 44 (14) | 31 (20) | 43 (21) |
| Other | 44 (14) | 31 (20) | 50 (25) |
| Unknown | 11 (10) | 16 (10) | 6 (3) |
| TOTAL | **314** | **153** | **201** |
