# Supplementary material for: The incidence, characteristics and outcomes of pregnant women hospitalized with symptomatic and asymptomatic SARS-CoV-2 infection in the UK from March to September 2020: A national cohort study using the UK Obstetric Surveillance System (UKOSS)
Source: PLoS One. 2021 May 5;16(5):e0251123. doi: 10.1371/journal.pone.0251123 (PMC8099130; doi:10.1371/journal.pone.0251123)
Supplement: S8 Table — (DOCX) [file pone.0251123.s008.docx]

**S8 Table. Hospital, pregnancy and infant outcomes amongst women with confirmed SARS-CoV-2 infection in pregnancy compared to a historical cohort without SARS-CoV-2 infection**

| Maternal outcomes | Women with SARS-CoV-2 (N=1148) | Historical comparison cohort (N=694) | OR (95% CI) | aOR (95% CI) |
| --- | --- | --- | --- | --- |
|  | **Number (%)** | **Number (%)** |  |  |
| Required critical care | 63 (5%) | 1 (<1%) | 40.18 (5.56-290.34)  p<0.001 | **33.50 (4.57-245.38)**  **p<0.001** |
| SARS-CoV-2 pneumonia | 173 (15%) | - | - | - |
| Pre-eclampsia | 20 (2%) | 8 (1%) | 1.52 (0.67-3.47)  p=0.320 | **1.15 (0.47-2.82)**  **p=0.767** |
| Died | 8 (<1%) | 0 | - | - |
| Ongoing pregnancy | 47 (4%) | 0 | - | - |
| Missing birth information** | 65 (6%) | 0 | - | - |
| Pregnancy known completed | 1036 (90%) | 694 (100%) | - | - |
|  |  |  |  |  |
| Pregnancy loss before 24 weeks’ of gestation | 31 (3%) | 2 (<1%) | NC | NC |
| Mode of birth |  |  |  |  |
| Pre-labor cesarean | 302 (30%) | 124 (18%) | 2.51 (1.96-3.23)  p<0.001 | **2.34 (1.77-3.10)**  **p<0.001** |
| Cesarean after labor onset | 165 (16%) | 77(11%) | 2.21 (1.63-2.99)  p<0.001 | **2.11 (1.51-2.95)**  **p<0.001** |
| Operative vaginal | 129 (13%) | 71 (10%) | 1.87 (1.36-2.58)  p<0.001 | **2.14 (1.51-3.05)**  **p<0.001** |
| Unassisted vaginal | 407 (41%) | 420 (61%) | 1 | **1** |
| Missing | 2 | 0 | - | - |
| Iatrogenic preterm birth <37 weeks’ | 105 (11) | 8 (1) | 10.05 (4.86-20.76)  p<0.001 | **9.70 (4.39-21.42)**  **p<0.001** |
| Spontaneous preterm birth <37 weeks’ | 44 (4) | 46 (7) | 0.65 (0.42-0.99)  p=0.045 | **0.63 (0.38-1.02)**  **p=0.059** |
|  | **Infant outcomes (N=1019)** | **Infant outcomes (N=705)** |  |  |
| Stillbirth | 9 (1%) | 2 (<1%) | 3.13 (0.68-14.55)  p=0.145 | **2.69 (0.53-13.66)**  **p=0.233** |
| Live birth | 1008 (99%) | 703 (100%) | 0.32 (0.07-1.48)  p=0.144 | **0.37 (0.07-1.88)**  **p=0.231** |
| Missing | 2 | 0 | - | **-** |
| Neonatal unit admission | 156 (15%) | 37 (5%) | 2.70 (1.91-3.83)  p<0.001 | **2.71 (1.80-4.01)**  **p<0.001** |
| Neonatal death | 4 (<1%) | 1 (<1%) | 2.81 (0.31-25.2)  p=0.355 | **5.30 (0.52-54.39)**  **p=0.160** |
| Gestation at birth (weeks)*** |  |  |  |  |
| 22-27 | 9 (1%) | 6 (1%) | 1.13 (0.40-3.20)  p=0.815 | **0.87 (0.24-3.12)**  **p=0.826** |
| 28-31 | 25 (2%) | 6 (1%) | 3.14 (1.28-7.70)  p=0.012 | **2.61 (1.01-6.75)**  **p=0.049** |
| 32-36 | 122 (12%) | 51 (7%) | 1.80 (1.28-2.54)  p=0.001 | **1.73 (1.18-2.53)**  **p=0.005** |
| 37 or more | 847 (84%) | 639 (91%) | 1 | **1** |
| Median (IQR) | 39 (38-40) | 40 (38-41) | **-** | **-** |
| Missing | 7 | 1 | **-** | **-** |

*Percentages of those with known infant outcome data (two women lost to follow up)

**EDD before 1^st^ October and no birth outcome available

*** excluding stillborn babies

NC – not comparable due to sources of data
